# Alcohol consumption and risk of dementia: A dose-response meta-analysis

**DOI:** 10.1097/MD.0000000000016099

**Published:** 2019-06-28

**Authors:** Jing Li, Xu Hui, Qinghong Gu, Yongfeng Lao, Zhenxing Lu, Lijuan Hou, Bibo Jia, Junqiang Niu, Liang Yao, Peijing Yan

**Affiliations:** a School of Public Health, Lanzhou University; b Evidence-Based Medicine Center of Lanzhou University; c Second Clinical Medical College of Lanzhou University; d First Clinical Medical College of Lanzhou University; e The First Hospital of Lanzhou University, Lanzhou; f Chinese Medicine Faculty of Hong Kong Baptist University, Kowloon Tong, Hong Kong; g Institute of Clinical Research and Evidence Based Medicine, Gansu Provincial Hospital, Lanzhou, China.

**Keywords:** alcohol, dementia, dose-response, meta-analysis, protocol

## Abstract

**Objective:**

To investigate the relationship between alcohol consumption and the incidence of dementia.

**Method:**

We will conduct a systematic search without language and year restrictions to identify all relevant published studies. The following electronic databases will be searched: PubMed, EMBASE, the Cochrane Library, Chinese BioMedical Literature Database (CBM) and China National Knowledge Infrastructure (CNKI), VIP, Wan-Fang. Cohort studies published in Chinese or English are considered for inclusion. Two authors will independently select studies base on inclusion criteria, extract data and assess the quality of included studies using the Newcastle–Ottawa Scale, the Grades of Recommendation, Assessment, Development, and Evaluation (GRADE) system will be used to quantify absolute effects and quality of evidence. Any disagreement will be resolved by consensus. We will use the hazard ratio (HR) as the effect indicator, piecewise linear regression model and restricted cubic spline model will be used for linear and nonlinear trend estimation, respectively.

**Registration:**

The dose-response meta-analysis is registered in the PROSPERO (CRD42019127367) international prospective register of systematic review.

**Discussion:**

In the previous related dose-response meta-analysis studies, there were some limitations: on the 1 hand, the sex was not taken into account. On the other hand, relative risk (RR) is not the best effect indicator for time-to-event data, but compare with RR, HR is much better. This study intends to use HR as the effect indicator to explore the dose-response relationship and the sex difference between alcohol intake and dementia. Accurate alcohol drinking data can provide high-quality evidence for the prevention of dementia.

## Abstract 2

Supplemental Digital Content is available in the text

## 1 Introduction

Dementia is 1 common neurodegenerative disease, it shows cognitive, behavioral, and psychological symptoms that can include memory loss, problems with reasoning and communication.^[1]^ Worldwide, 50 million people live with dementia, and this number will increase to approximately more than 152 million by 2050, leading to a huge burden.^[2]^ Because most dementias are not curable, the prevention of dementia has been increasingly catching attention of the world.^[3]^

As a globally consumed beverage, alcohol is one of the modifiable risk factors of dementia.^[4,5]^ However, some studies showed light-to-moderate alcohol intake may protect against dementia.^[6–8]^ There was a meta-analysis in 2016 quantifying the dose-response between alcohol consumption and dementia.^[4]^ However, this study did not consider sex differences. Dementia is usually greater among women than men, and many studies considered sex separately.^[3,9–11]^ Moreover, the use of relative risk (RR) as merge indicator might result in a loss of information about time-to-event data in studies.^[5,11,12]^ Additionally, most of the published related researches in recent years was time-to-event data, and the hazard ratio (HR) is more appropriate for time-to-event data and used widely.^[8,13,14]^ High-quality meta-analysis has been increasingly regarded as one of the key tools for achieving evidence.^[15,16]^ Therefore, we use HR as the effect indicator to do a meta-analysis to explore whether there is a dose-response relationship between alcohol consumption and dementia.

## 2 Method

### 2.1 Registration

This dose-response meta-analysis had applied for registration in the PROSPERO (CRD42019127367) international prospective register of systematic review. Following the proposals by the Preferred reporting items for systematic review and meta-analysis protocols (PRISMA-P) statements, to report the dose-response meta-analysis (DRMA).^[17–20]^ A MeaSurement Tool to Assess Systematic Reviews (AMSTAR) will be used to assess methodological quality.^[21,22]^ The Grades of Recommendation, Assessment, Development, and Evaluation (GRADE) system will be used to quantify absolute effects and quality of evidence.^[23,24]^ No requirement of ethical approval and informed consent is needed because it's a retrospective study.

### 2.2 Search strategy

We will conduct a systematic search without language and year restrictions to identify all relevant published studies. The following electronic databases will be searched: PubMed, EMBASE, and the Cochrane Library, Chinese BioMedical Literature Database (CBM) and China National Knowledge Infrastructure (CNKI), VIP, Wan-Fang. The search strategy will include terms relating to the intervention (alcohol) and the disease (dementia) (Supplementary file-1). References of included study will also be traced back to find potential qualified studies. We will contact the authors if the included articles were not fully reported.

### 2.3 Selection criteria

Studies will be included if they met the following criteria:

(1) cohort studies;
(2) investigated the association between dementia (diagnosed by DSM-5) and alcohol intake^[3]^;
(3) alcohol intake was categorized into ≥3 levels, and drinking levels can be quantified;
(4) acquired directly or indirectly about level-specific HR/RR/and 95% confidence interval (CI).

If any of the following conditions are met, the study will be excluded:

(1) reviews, comments, letters, conference abstracts, editorials, systematic reviews or meta-analyses, protocols, case reports, case–control studies, cross-section studies, nonhuman studies, case series, briefs, and so on;
(2) the outcome is Korsakoff's syndrome, Webster's disease, Parkinson's disease, Mild cognitive impairment (MCI), and so on;
(3) studies with incomplete data;
(4) Not in Chinese or English.

Additionally, if multiple articles were published based on the same cohort, we chose that with longer follow-up or a larger sample size.

### 2.4 Data extraction and quality evaluation

The 2 researchers will independently screen literatures, conduct data extraction, and quality evaluation. Any disagreement regarding inclusion will be resolved by discussion among all review authors. Researchers will screen titles and abstracts of the studies base on inclusion/exclusion criteria, and select the full text of potentially relevant studies for further assessment.

A standardized form will be used to extract data from the included studies. From each study, we extracted the first author, publication year, region, sex, age, follow-up duration, sample size and person-years stratified by alcohol dose, diagnosis criteria, alcohol intake, HR /RR and 95% CI.

#### 2.4.1 Quality evaluation

The study quality will be evaluated with the Newcastle-Ottawa Quality Assessment Scale (NOS).^[25]^ The NOS contains 8 items, categorized into 3 dimensions including selection, comparability, and outcome (cohort studies). A star rating system will be used to semi-quantitatively evaluate the quality of the study, which allows a total star of up to 9 and studies with more than 6 stars will be evaluated as high quality.

### 2.5 Statistical analysis

The meta-analysis will be conducted using STATA version 12.0 (STATA Corp, College Station, TX). Piecewise linear regression model and restricted cubic spline (RCS) model will be used for nonlinear trend estimation, a flexible meta-regression based on RCS function will be used to fit the potential nonlinear trend, and generalized least-square method will be used to estimate the parameters.^[26]^ The HR and 95% CI will be used as the pooled effect size for time-to-event data, and RR and 95% CI will be used as the effect indicator for other dichotomy variables.

Q test and I^2^ statistic will be used to evaluate heterogeneity of merged studies. I^2^ >50% and *P* <.05 is defined as a significant heterogeneity. When significant heterogeneity exists, a random-effects model will be used; otherwise, the fixed-effect model will be used. If there is a high heterogeneity between studies, subgroup analysis, and sensitivity analysis will be used to explore heterogeneity.

#### 2.5.1 Subgroup analysis and sensitive analysis

Subgroup analysis will be conducted base on different clinical subgroups below:

(1) sex of patients;
(2) study types (prospective and retrospective studies);
(3) presence or absence of Apolipoprotein e4 allele.

Sensitivity analysis will be carried out by excluding trials and results will be presented and compared with overall findings.

## 3 Discussion

To the best of our knowledge, meta-analysis is one of the main sources of high-quality evidence,^[16]^ especially the DRMA have higher quality,^[18]^ this makes up for some shortcomings of the original study such as the inconsistency in the unit of alcohol measurement and definition of light, moderate and severe drinking. 60% of the original studies included in previous DRMA were time-to-event data.^[4]^ For time-to-event data, the use of RR results in loss of data, leading to erroneous conclusions. Our study intends to use HR as the effect indicator, which is more appropriate for time-to-event data. Additionally, we will conduct subgroup analyses to explore the dose-response relationship between alcohol and dementia in different sexes and study types.

However, there are several limitations and difficulties in our study. First, different definitions of alcohol consumption may lead to heterogeneity. Second, we cannot exclude the potential influences of including former drinkers, who may quit drinking due to underlying diseases and have a high risk of dementia, in the reference group due to data restrictions. Another limitation is considerations of the inconsistence of the adjusted confounders in included studies; we cannot exclude the potentially spurious inverse association caused by some confounders.

In this context, the subgroup analysis about dementia types can’t be conduct because of the lack of the sample size. In future, we will explore the dose-response relationship between alcohol and dementia in different dementia types, alcohol measurements, and definitions.

## Author contributions

All authors approved the final version of the manuscript.

**Conceptualization:** Xu Hui, Liang Yao, Peijing Yan, Jing Li.

**Data curation:** Jing Li, Xu Hui, Qinghong Gu, Zhenxing Lu, Lijuan Hou, Bibo Jia, Junqiang Niu, Peijing Yan.

**Formal analysis:** Jing Li, Xu Hui, Qinghong Gu, Liang Yao.

**Investigation:** Jing Li, Yongfeng Lao, Lijuan Hou, Liang Yao.

**Methodology:** Jing Li, Xu Hui, Qinghong Gu, Yongfeng Lao, Peijing Yan.

**Project administration:** Liang Yao, Peijing Yan.

**Software:** Xu Hui, Qinghong Gu, Zhenxing Lu, Peijing Yan.

**Supervision:** Peijing Yan, Liang Yao.

**Writing – original draft:** Jing Li, Xu Hui, Qinghong Gu.

**Writing – review & editing:** Yongfeng Lao, Zhenxing Lu, Lijuan Hou, Bibo Jia, Junqiang Niu, Liang Yao, Peijing Yan.

## Supplementary Material

Supplemental Digital Content
