# Supplementary material for: Alcohol consumption and risk of dementia: A dose-response meta-analysis
Source: Medicine (Baltimore). 2019 Jun 28;98(26):e16099. doi: 10.1097/MD.0000000000016099 (PMC6617082; doi:10.1097/MD.0000000000016099)
Supplement: Supplemental Digital Content [file medi-98-e16099-s001.docx]

**Part of the search strategy is as follows:**

**1.EMBASE**

**#1 'alcohol'/exp OR 'alcohol blood level'/exp OR 'drinking behavior'/exp OR 'alcohol consumption'/exp**

**#2 'ethanol':ab,ti OR 'alcohol*':ab,ti OR 'blood alcohol level':ab,ti OR 'blood alcohol content':ab,ti OR 'wine*':ab,ti OR 'liquor*':ab,ti OR 'spirit*':ab,ti OR 'beer*':ab,ti OR 'beverage*':ab,ti OR 'alcohol drinking':ab,ti OR 'drinking behavior':ab,ti OR 'alcohol consumption':ab,ti OR 'alcohol intake':ab,ti OR 'drink*':ab,ti OR 'drunk*':ab,ti**

**#3 #1 OR #2**

**#4 'dementia'/exp OR 'alzheimer disease'/exp OR 'cadasil'/exp OR 'diffuse lewy body disease'/exp OR 'frontotemporal dementia'/exp OR 'hiv associated dementia'/exp OR 'huntington chorea'/exp OR 'multiinfarct dementia'/exp**

**#5 'parkinson disease'/exp OR 'delirium'/exp OR 'cognitive defect'/exp**

**#6 'dement*':ab,ti OR 'vascular dementia':ab,ti OR 'frontotemporal dementia':ab,ti OR 'alzheimer*':ab,ti OR 'lewy body':ab,ti OR 'lewy bodies':ab,ti OR 'huntington*':ab,ti OR 'parkinson*':ab,ti OR 'deliri*':ab,ti OR 'neurocognitive disorder':ab,ti OR 'cognitive disorder':ab,ti OR 'multiinfarct dementia':ab,ti OR 'multi-infarct dementia':ab,ti OR 'cadasil':ab,ti OR 'aids dementia complex':ab,ti OR 'hiv associated dementia':ab,ti OR 'aphrenia':ab,ti OR 'cognitive defect':ab,ti OR 'cognitive impairment':ab,ti OR 'age-associated memory impairment':ab,ti OR 'cognitive disorders':ab,ti OR 'neurocognitive disorders':ab,ti OR 'cognitive impairments':ab,ti OR 'age-associated memory impairments':ab,ti**

**660,800**

**#7 #4 OR #5 OR #6**

**#8 #3 AND #7**

**2.CBM**

#1 ((((("阿尔茨海默病"[不加权:扩展])) OR "痴呆"[不加权:扩展]) OR "痴呆, 血管性"[不加权:扩展]) OR "痴呆, 多发性梗死性"[不加权:扩展]) OR "认知障碍"[不加权:扩展]

#2 (((((((("痴呆"[常用字段:智能]) OR "痴呆，血管性"[常用字段:智能]) OR "痴呆，多发性梗死性"[常用字段:智能]) OR "阿尔茨海默病"[常用字段:智能]) OR "认知障碍"[常用字段:智能]) OR "路易体痴呆"[常用字段:智能]) OR "血管性痴呆"[常用字段:智能]) OR "帕金森病痴呆"[常用字段:智能]) OR "认知损伤"[常用字段:智能]

#3 (#2) OR (#1)

#4 "饮酒"[不加权:扩展]

#5 ((("酒精"[常用字段:智能]) OR "饮酒"[常用字段:智能]) OR "喝酒"[常用字段:智能]) OR "酒"[常用字段:智能]

#6 (#5) OR (#4)

#7 (#6) AND (#3)
